# Withdrawal: Selective cleavage of BLM, the Bloom syndrome protein, during apoptotic cell death.

**DOI:** 10.1074/jbc.W120.016689

**Published:** 2021-01-13

**Authors:** Oliver Bischof, Sanjeev Galande, Farzin Farzaneh, Terumi Kohwi-Shigematsu, Judith Campisi

VOLUME 276 (2001) PAGES 12068–12075

This article has been withdrawn by the authors except Dr. Kohwi-Shigematsu, who could not be reached. Fig. 3*B* has a duplication of the top band in *lanes 1* and *3*. Fig. 5*D* has a duplication between the top bands and a horizontal flip of the bottom bands. Fig. 5*E* likewise has a duplication of the top bands excluding the probe lanes and a horizontal flip of the bottom bands. Fig. 6*A* has smaller microscopy images pasted on the background of larger microscopy images.

